# Monitoring brain development of chick embryos in vivo using 3.0 T MRI: subdivision volume change and preliminary structural quantification using DTI

**DOI:** 10.1186/s12861-015-0077-6

**Published:** 2015-07-25

**Authors:** Zien Zhou, Zengai Chen, Jiehui Shan, Weiwei Ma, Lei Li, Jinyan Zu, Jianrong Xu

**Affiliations:** Department of Radiology, Ren Ji Hospital, School of Medicine, Shanghai Jiao Tong University, Shanghai, China; Department of Geriatrics, Ren Ji Hospital, School of Medicine, Shanghai Jiao Tong University , Shanghai, China

**Keywords:** Chick embryo, Brain development, In vivo, Magnetic resonance imaging, Diffusion tensor imaging

## Abstract

**Background:**

Magnetic resonance imaging (MRI) has many advantages in the research of in vivo embryonic brain development, specifically its noninvasive aspects and ability to avoid skeletal interference. However, few studies have focused on brain development in chick, which is a traditional animal model in developmental biology. We aimed to serially monitor chick embryo brain development in vivo using 3.0 T MRI.

**Methods:**

Ten fertile Hy-line white eggs were incubated and seven chick embryo brains were monitored in vivo and analyzed serially from 5 to 20 days during incubation using 3.0 T MRI. A fast positioning sequence was pre-scanned to obtain sagittal and coronal brain planes corresponding to the established atlas. T2-weighted imaging (T2WI) was performed for volume estimation of the whole brain and subdivision (telencephalon, cerebellum, brainstem, and lateral ventricle [LV]); diffusion tensor imaging (DTI) was used to reflect the evolution of neural bundle structures.

**Results:**

The chick embryos’ whole brain and subdivision grew non-linearly over time; the DTI fractional anisotropy (FA) value within the telencephalon increased non-linearly as well. All seven scanned eggs hatched successfully.

**Conclusions:**

MRI avoids embryonic sacrifice in a way that allows serial monitoring of longitudinal developmental processes of a single embryo. Feasibility for analyzing subdivision of the brain during development, and adding structural information related to neural bundles, makes MRI a powerful tool for exploring brain development.

## Background

Several imaging techniques have been developed to assess embryonic development among small animals, including special light microscopy (confocal fluorescence microscopy, multi-photon microscopy, optical coherence tomography), ultrasound, micro-CT, micro-MRI, etc. [1–5]. Ideally, in vivo embryonic research should avoid embryonic sacrifice, which allows monitoring of a serial, longitudinal developmental process of a single embryo. MRI has great advantages over other techniques in the research of in vivo embryonic brain development given its high spatial and tissue-contrast resolution, no skull interference, flexible imaging plane orientation, and absence of radiation and obvious teratogenical influences. Diffusion tensor imaging (DTI) tends to reflect aspects of tissue structure by detecting water molecule diffusion through those tissues. Fractional anisotropy (FA) is a DTI-derived measurement of tissue structural anisotropy ranging from 0 (isotropy) to 1 (anisotropy) [6]. DTI could be used to reflect brain maturation by measuring diffusion characteristics based on neuronal differentiation and neural bundle formations [7–9]. For instance, mesenchymal cells or neuroblasts are round in shape, allowing water to diffuse isotropically (FA is approximately 0). Conversely, mature neural bundles have a fiber-like texture, and water molecules mainly diffuse along the bundle’s direction (FA is approximately 1).

In vivo MRI studies assessing normal mouse, baboon, and marmoset brain development have been conducted [10–12]. However, there are few in vivo MRI studies assessing serial brain development of the chick embryo. The chick embryo is an accessible and economical model, with an extensive history of use in developmental biology, genetic experiments, transplantation research, teratogenicity evaluation, and cancer research [13, 14]. The brain developmental time frame for chicks is also similar to humans, with both chick and human newborns showing well developed brains at hatching and birth, respectively [15]. Bain et al. [16] estimated the brain volume of developing chick embryos using a 7.0 T MRI system, but images were only obtained at late developmental stages (12, 15, 17, 18, 19, 20 days during incubation). Thus, some problems are encountered and need to be resolved when assessing in vivo MRIs of embryonic chick brains. (a) Embryo-associated motion artifacts seriously reduce imaging quality after 6 days of incubation. Several methods have been proposed to effectively suppress motion artifacts, including the use of a fast imaging sequence, intravenous injection of a small-dose sedative or anesthetic [17], and cooling before imaging [18]. (b) In order to balance long scan times, the spatial resolution and signal-to-noise ratio (SNR) for in vivo imaging should be set relatively lower than for ex vivo imaging. However, the spatial resolution and SNR need to be increased sufficiently to account for small embryos during the early developmental stages. Micro-MRIs with ultra-high magnetic fields (≥7 T) and a dedicated small coil that is able to fit a small embryo are commonly adopted. (c) Because embryonic position is variable, it is almost impossible to obtain axial, sagittal, and coronal brain slices directly after scanning. Images with a 3D MRI sequence have high isotropic spatial resolution and could be used to reconstruct standard axial, sagittal, and coronal slices corresponding to an established atlas during post-processing. However, this is sometimes a less attractive option for in vivo research because of long scan durations [19]. Additionally, some forms of functional and structural imaging (such as magnetic resonance spectroscopy [MRS], blood oxygen level dependent MRI [BOLD], diffusion weighted imaging [DWI], DTI, etc.) do not have a 3D sequence. Thus, a 2D sequence is adopted. When obtaining 2D images with high resolution, adjusting the positioning lines and using a fast positioning sequence with a large field of view (FOV) and thick slice are usually needed to obtain axial, sagittal, and coronal slices (which often requires repeated attempts).

The present study aimed to serially monitor chick embryo brain development in vivo using 3.0 T MRI from 5 to 20 days of incubation. A method of adjusting the positioning lines was proposed using 2D imaging to obtain standard sagittal and coronal slices corresponding to an established atlas [20]. Whole brain and subdivision (telencephalon, cerebellum, brainstem, and lateral ventricle [LV]) volume changes were measured from anatomical images on a day-to-day basis. The DTI FA value was preliminarily used to reflect structural evolution in telencephalon.

## Results

**Fig. 1:**
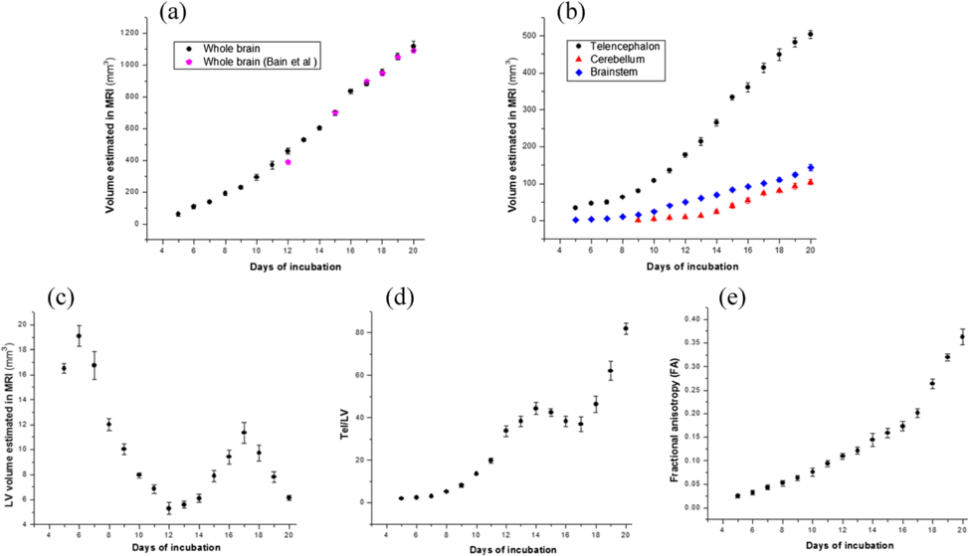
The growth plots of chick embryonic brain from 5 to 20 days of incubation (*n* = 7, mean ± SD). **a** The MRI volume estimates of the whole brain and the data reported by Bain et al.; **b** the MRI volume estimates of telencephalon, cerebellum and brainstem; **c** the MRI volume estimates of LV; **d** the change of Tel/LV ratio; **e** the change of DTI FA value in telencephalon

All seven scanned eggs were normally developing from 5 to 20 days of incubation (eggs were candled before MR scanning and capillary network was monitored to identify the developing status), and hatched successfully after 23 days of incubation. All images of the seven scanned eggs had no obvious motion artifacts and were adopted for analysis. Table 1 shows the whole brain volume, subdivision volume (telencephalon, cerebellum, brainstem, and LV), volume ratio of telencephalon to lateral ventricle (Tel/LV), and the DTI FA value from 5 to 20 days of incubation. The cerebellum could not be clearly discriminated until 9 days of incubation; thus, we measured the cerebellum from 9 to 20 days of incubation. Fig. 1 plots corresponding growth change over time. The whole brain, telencephalon, cerebellum, and brainstem grew non-linearly during incubation. The whole brain volume reported by Bain et al. is also shown in Fig. 1a for comparison. LV volume did not decrease linearly over time, and flex points appeared at 6, 12, and 17 days of incubation. The Tel/LV ratio did not increase linearly over time, and flex points appeared at 14 and 17 days of incubation. The FA value of the telencephalon increased non-linearly from 5 days (0.026 ± 0.004) to 20 days (0.362 ± 0.017).

**Table 1:** The data of whole brain volume, subdivision volume (telencephalon, cerebellum, brainstem, and LV), volume ratio of the telencephalon to the lateral ventricle (Tel/LV), and the DTI FA value from 5 to 20 days of incubation. The structure of cerebellum could not be discriminated until 9 days of incubation in our images

Days	Volume, mm^3^ ( ± SD)					Tel/LV ( ± SD)	FA ( ± SD)
	Whole brain	Telencephalon	Cerebellum	Brainstem	LV		
D_5	60.08 (10.14)	33.33 (2.95)	-	1.98 (0.38)	16.51 (0.38)	2.02 (0.15)	0.026 (0.004)
D_6	105.97 (11.75)	46.02 (1.83)	-	3.65 (0.48)	19.09 (0.83)	2.41 (0.10)	0.032 (0.005)
D_7	136.66 (8.77)	50.32 (5.22)	-	5.96 (0.54)	16.77 (1.11)	3.00 (0.24)	0.043 (0.005)
D_8	189.68 (10.77)	62.90 (3.46)	-	10.64 (1.18)	12.01 (0.47)	5.24 (0.28)	0.052 (0.006)
D_9	228.02 (10.80)	80.22 (4.81)	2.04 (0.16)	16.23 (2.45)	10.03 (0.44)	8.02 (0.73)	0.063 (0.005)
D_10	292.02 (18.15)	107.47 (2.70)	4.42 (0.20)	24.32 (1.32)	7.95 (0.20)	13.53 (0.37)	0.076 (0.009)
D_11	369.73 (26.31)	135.22 (6.33)	7.84 (0.60)	40.28 (2.53)	6.85 (0.33)	19.78 (1.31)	0.094 (0.007)
D_12	458.48 (17.68)	177.98 (6.70)	10.68 (0.34)	49.99 (3.12)	5.30 (0.49)	33.77 (2.55)	0.109 (0.006)
D_13	527.99 (9.73)	214.38 (10.75)	14.77 (1.75)	60.42 (3.13)	5.61 (0.29)	38.27 (2.38)	0.121 (0.007)
D_14	603.38 (11.35)	265.40 (8.50)	25.15 (3.05)	69.46 (4.92)	6.11 (0.33)	44.33 (2.87)	0.144 (0.014)
D_15	695.89 (14.37)	333.62 (6.17)	39.80 (6.72)	83.68 (5.00)	7.88 (0.47)	42.43 (1.86)	0.159 (0.010)
D_16	835.56 (16.17)	360.58 (12.68)	53.89 (7.14)	92.65 (3.45)	9.42 (0.56)	38.38 (2.41)	0.173 (0.010)
D_17	881.36 (11.36)	413.23 (11.92)	73.18 (4.77)	101.38 (3.17)	11.35 (0.84)	36.98 (3.37)	0.201 (0.009)
D_18	956.55 (16.40)	449.41 (16.15)	80.49 (4.14)	110.66 (5.72)	9.73 (0.62)	46.36 (3.87)	0.264 (0.010)
D_19	1053.39 (21.09)	482.43 (12.11)	93.48 (7.94)	123.27 (5.16)	7.79 (0.43)	62.10 (4.48)	0.320 (0.007)
D_20	1117.96 (31.45)	503.41 (10.79)	104.58 (6.82)	143.77 (8.58)	6.15 (0.22)	81.91 (2.56)	0.362 (0.017)

**Fig. 2:**
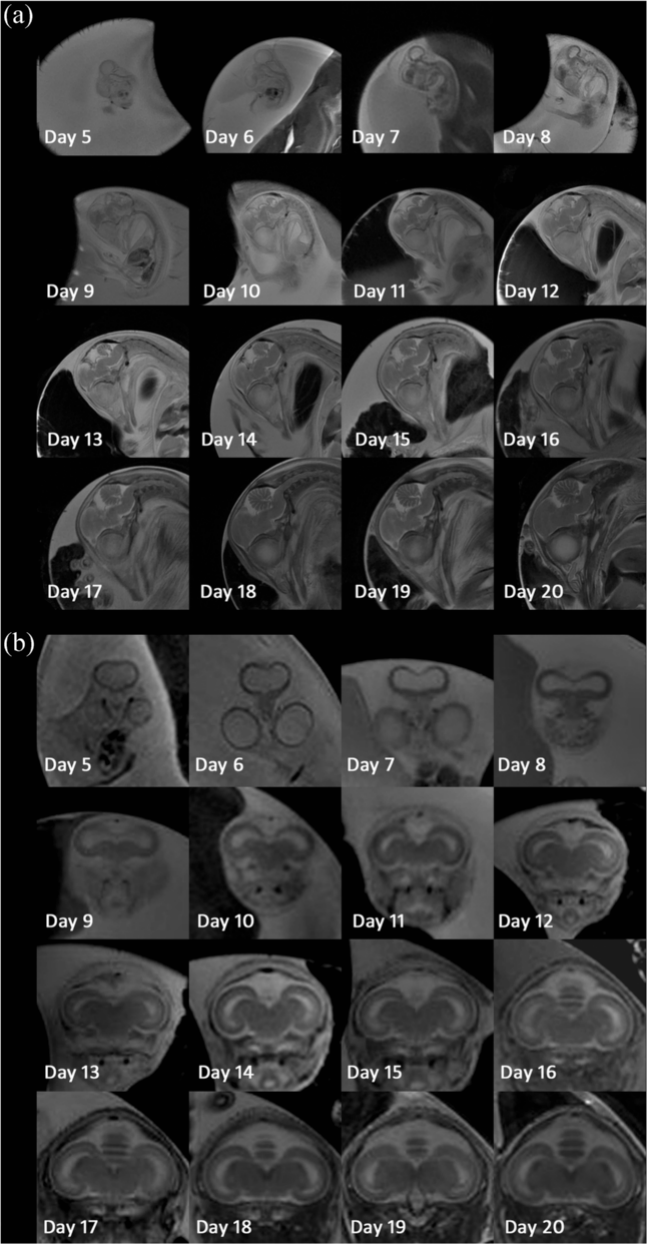
The brain developing process serially of one chick embryo from 5 to 20 days of incubation. **a** In the mid-sagittal plane of the brain; **b** in the coronal plane with the maximum ventricle area

Figure 2 shows serial brain developmental processes for one chick embryo in the mid-sagittal and coronal planes with a maximum ventricle area. As shown in Fig. 2a, the telencephalic vesicles were formed at an early developmental stage. The telencephalon, cerebellum, and brainstem could be discriminated at 9 days and grew gradually over the following incubation days. As shown in Fig. 2b, the parenchyma was very thin, and the ventricle was relatively large at early developmental stages. The cortex became thicker, and the ventricle was reduced over the course of incubation.

## Discussion

We serially monitored chick-embryo brain development in vivo using 3.0 T MRI. Compared with previous work, we first conducted serial scans across an early to late developmental stage (5 to 20 days of incubation) at a constant interval (24 h). Bain et al. [16] only scanned during the late stage (12 to 20 days of incubation) at a non-constant interval. To our knowledge, in vivo MR imaging of chick embryonic brain development prior to 5 days post-incubation has not been reported. Hogers et al. [19] noninvasively monitored chick embryo cardiovascular development at 3, 5, 7, 9, and 11 days of incubation using 9.4 T MRI. However, the structure of cephalic vesicles could not be clearly discriminated at 3 days in their study given the SNR. Embryonic sagittal and coronal slices corresponding to an established atlas were obtained in the present study and the imaging planes were nearly identical in orientation over time; this was not implemented in Bain et al.’s study. Thus, different anatomical structures could easily be distinguished based on an established atlas. Changes within the same structure at different developmental stages could be monitored among corresponding slices. Furthermore, changes in subdivision (telencephalon, cerebellum, brainstem, lateral ventricle, or cephalic vesicles) volume over time and DTI structural evolution in the telencephalon were measured in the present study. These methods were yet to be utilized and could not be implemented using histological methods. We used a clinical 3.0 T MR system, which is more widely available and includes a lower specific absorption rate and shorter scan duration compared with a 7.0 T or higher system. Our T2-weighted image in-plane resolution (172 μm for sagittal planes and 195 μm for coronal planes) and image quality were not overly inferior to those obtained with a 7.0 T or higher system (195 μm in Bain et al.’s study using a 7.0 T system [16] and 78–90 μm in Hogers et al.’s study using a 9.4 T system [19]), which implies that a 3.0 T system is feasible for anatomical analysis of embryonic chick brain development in vivo.

Our whole brain volume data at 15 (695.89 ± 14.37 mm^3^), 17 (881.36 ± 11.36 mm^3^), 18 (956.55 ± 16.40 mm^3^), 19 (1053.39 ± 21.09 mm^3^), and 20 (1117.96 ± 31.45 mm^3^) days of incubation is nearly consistent with Bain et al.’s data; however, we noted larger brain volume at 12 days of incubation (458.48 ± 17.68 mm^3^ vs about 400 mm^3^) as shown in Fig. 1a. At the early and middle developmental stages, the cerebral cortex and white matter are immature, and the brain contains a large amount of cerebrospinal fluid. In our segmentation, the whole brain included part cerebrospinal fluid and part cerebral parenchyma, which might explain why our whole brain volumes were larger than in Bain et al.’s study at 12 days of incubation. In our study, the whole brain, telencephalon, cerebellum, and brainstem volumes of chick embryos increased non-linearly over time. This non-linear growth pattern is also supported by Goedbloed et al.’s and Habas et al.’s finding in rat, mouse and human fetus [21, 22]. LV volume and the Tel/LV ratio increased or decreased at flex points. In our opinion, the LV volume change during development depends on two factors: increasing brain volume and increased cortical thickness. Although both of these factors increased over time, one of these factors may have been more dominant at a certain stage. Based on the LV volume change curve and Tel/LV ratio shown in Fig. 1c and d, increasing cortical thickness mainly occurred at early (5 to 13 days of incubation) and late (18 to 20 days) developmental stages. This led to LV volume decreases and Tel/LV ratio increases. During the middle developmental stage (14 to 17 days), the increasing brain volume level exceeded that of cortical thickness, which led to an LV volume increase and Tel/LV ratio decrease.

In addition to the anatomical information obtained from traditional T1- and T2-weighted images, functional and structural information could also be reflected in MR images. Cahill et al. [23] showed blood redistribution among fetal mice during hypoxia using BOLD MRI. BOLD signal intensity decreased by 12 ± 7 % in the fetal brain and by 44 ± 8 % in the liver as the maternal inspired gas mixture varied from hyperxia to hypoxia. This shows that blood flow is redistributed to preserve brain oxygenation at the expense of other organs. Peebles et al. [24] used MRS and DWI to reflect metabolic and diffusion conditions before, during, and after hypoxia among chick embryos in vivo. In our previous work [25, 26], we used DTI to monitor chick embryonic skeletal and smooth muscle development. The DTI FA value had a positive correlation with the quantitative histological analysis, which shows that the FA value could be used to measure fiber-like tissue maturation. In the present study, we extended to neural bundles and used the FA value to measure structural evolution. As shown in Fig. 1e, the average DTI FA value in the telencephalon non-linearly increased from 0.026 ± 0.004 (5 days) to 0.362 ± 0.017 (20 days). The FA value also showed a greater increase after 17 days of incubation than from 5 to 16 days. Skeletal and smooth muscle fibers could be tracked using DTI in our previous studies; however, we could not track the neural bundles. Liu et al. [11] used DTI to reflect fetal baboon brain maturation using 3.0 T MRI. Only mean diffusivity was measured in the white and grey matter, and no fibers were tracked. The low spatial resolution of 3.0 T diffusion imaging may be too limiting for fiber tracking.

All seven scanned eggs hatched successfully. However, the influence of MRI’s biological safety regarding chick embryonic development was not investigated in the present study. Other previous studies revealed that high external magnetic fields, gradient fields, and radio-frequency pulses had no apparent adverse effect on chick or quail embryonic development [18]. Cooling eggs before scanning to avoid embryonic-associated motion artifacts had no apparent adverse effect as well, which is noninvasive and often adopted for in vivo MR imaging of chick embryo. We should also note some limitations to the present study. For instance, we manually defined the borders between different brain subdivisions according to an established atlas. These borders are usually three-dimensional surfaces moving in and out of the imaging planes, which may induce subdivision volume measurement errors. Higher spatial resolution and thinner imaging slices may be helpful for more accurate measurement. However, image SNRs would reduce as a result. Nevertheless, the present study reveals the possible feasibility of using the DTI FA value to reflect neural bundle structural evolution. As this was a preliminary in vivo imaging study, no histological validation was conducted. Thus, a correlation analysis between the imaging parameters and histological quantification will be pursued in the future.

## Conclusions

The present study succeeded in serially monitoring chick embryonic brain development in vivo. The current methods included segmenting sagittal and coronal planes (corresponding to an established atlas) using our proposed positioning method. A 3.0 T MRI system is feasible for anatomical analysis of in vivo chick embryonic brain development; however, one caveat is that very early developmental stages could not be properly assessed. The present volume results were the first phase of quantitative analyses regarding brain subdivision development, which could not be implemented using an invasive histological method. Chick embryonic brain and subdivision growth patterns were non-linear. Additionally, the DTI FA value was first used as a measure of neural bundle maturation, which revealed structural evolution in the absence of resolution at the cellular level. Overall, this form of MRI has great potential for research on brain development.

## Methods

### Animals and treatments

The study was designed and implemented according to the ARRIVE guidelines [27]. For chick embryos represent an intermediate stage between isolated cultured cells and animals, animals’ housing and husbandry conditions listed in the ARRIVE guidelines checklist did not involve in our expreriment. The Animal Ethics Committee of Shanghai Jiao Tong University School of Medicine (No.20140125) approved this study. Ten fertile Hy-line white eggs, each weighting 50–55 g, were obtained from a commercial hatchery and placed in an incubator with automatic temperature (37.8 °C) and humidity (60 %) control. Eggs were automatically rolled every 2 h. After four days of incubation, eggs were candled with a hand-held light source to observe whether they were developing normally. We considered the chick embryo to be developing normally if the capillary network was observed. Three eggs were removed from the incubator due to underdevelopment or lack of fertility. The remaining seven eggs were scanned every 24 h from 5 to 20 days of incubation. Embryonic-associated motion artifacts are present at 7 days of incubation and lead to bad image quality. A dual-cooling method, as proposed in our previous work, was used to avoid motion artifacts [28]; the egg was cooled for 1 h in a refrigerator (at 3.5–4 °C) prior to imaging. During imaging, the egg was wrapped in a piece of Techni-Ice (Techni Ice, Victoria, Australia) to maintain a low-temperature environment.

### MR imaging and section method

MRI scans were performed using a 3.0 T GE Signa Excite System (GE Medical Systems, Waukesha, WI, USA) with a four-channel dedicated animal coil. The inner diameter of the coil was 5 cm, which precisely fits to an egg with wrapped Techni-Ice. First, fast positioning imaging was performed using a T2-weighted fast spin echo (FSE) sequence with a large FOV and thick slice (TR/TE 1763/43 ms, FOV 10 cm, matrix 320 × 320, ETL 1, NEX 1, Slice thickness 2 mm, gap 1 mm, No. of slices 8, 43-s duration) to obtain standard axial, sagittal, and coronal brain planes corresponding to an established atlas. Next, anatomical imaging of the sagittal and coronal brain planes was conducted using a T2-weighted FSE sequence at a higher resolution (Sagittal planes: TR/TE 4800/91 ms, FOV 5.5 cm, matrix 320 × 320, ETL 18, NEX 6, Slice thickness 1 mm, no gap, No. of slices 16, approximately 8-min duration; Coronal planes: TR/TE 4400/88 ms, FOV 5 cm, matrix 256 × 256, ETL 18, NEX 6, Slice thickness 1 mm, no gap, No. of slices 16, approximately 6-min duration). Finally, a DTI was performed, and the adjusted positioning lines for sagittal brain planes were adopted. The DTI parameters are as follows: 16 directions of diffusion gradients, TR/TE 5700/92 ms, FOV 8 cm, matrix 64 × 64, ETL 1, NEX 3, Slice thickness 1 mm, no gap, No. of slices 7, b = 800 s/mm^2^, approximately 6-min duration.

**Fig. 3:**
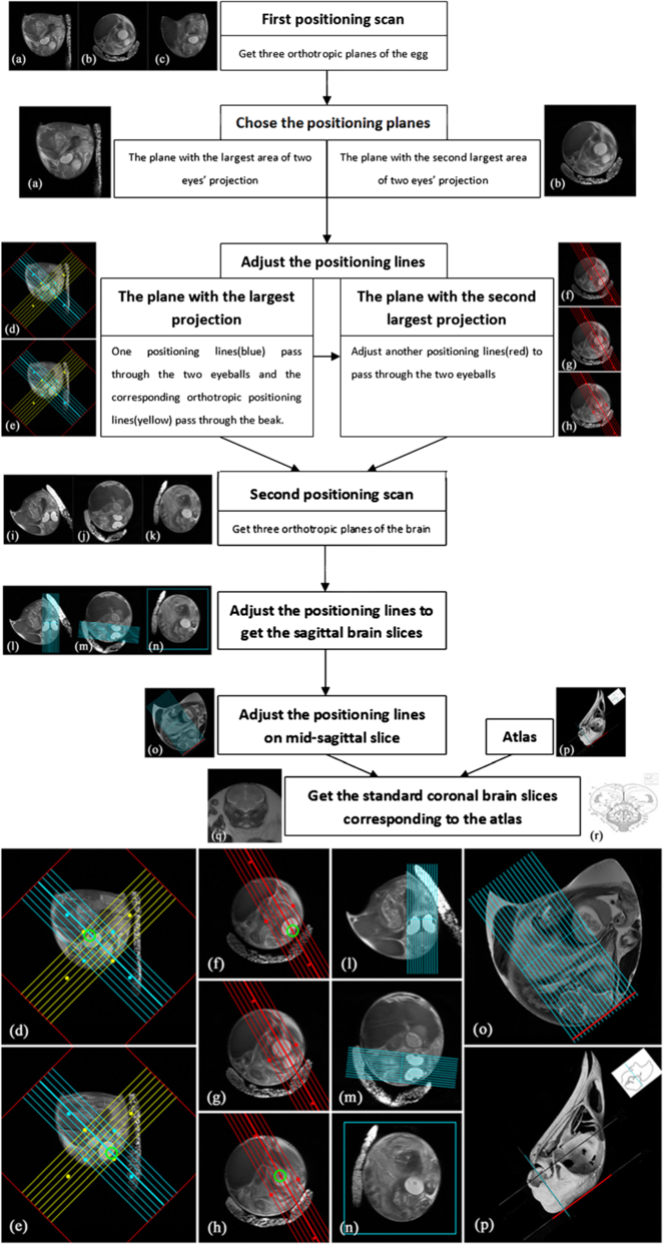
The flow chart and illustrations of our method to obtain the standard chick embryonic brain slices corresponding to the atlas in vivo. The amplificatory illustrations are shown in the bottom for clearly showing the positioning method. **a**, **b** and **c** are three orthotropic planes of the egg after first positioning scan; **d** and **e** are two serial slices in the plane with the largest area of two eyes’ projection. One set of positioning lines (blue) is adjusted to pass through the two eyeballs (green circle) and the corresponding orthotropic set of positioning lines (yellow) is put to pass through the beak; **f**, **g** and **h** are three serial slices in the plane with the second largest area of two eyes’ projection. Another set of positioning lines (red) should also be adjusted to pass through two eyeballs (green circle); **i**, **j** and **k** are three orthotropic planes of chick embryonic brain after second positioning scan; **l**, **m** and **n** show the positioning method to get the standard sagittal planes of brain with the positioning lines passing through the mid-line of the brain; **o** is the mid-sagittal plane with positioning lines corresponding to the section direction of atlas in **p**. The positioning lines to get coronal slices are perpendicular to the line which is tangent to the dorsal surface of parietal and frontal bones (red line in **o** and **p**). **q** is one of the standard coronal slices and **r** is corresponding established atlas [20]

Two eyeballs and the beak were used as the anatomical feature points when adjusting the positioning lines. Figure 3 shows the flow chart and illustrations of the positioning method. Because the chick embryonic position in the egg was unknown, after the first positioning scan (axial, sagittal, and coronal planes of the egg), three arbitrary orthotropic brain planes were obtained. We chose the plane with the largest area of eye projection, and the plane with the second largest eye projection was used as the base of the next positioning scan. We first adjusted one set of positioning lines to pass through two eyeballs in the plane with the largest projection, and a corresponding orthotropic set of positioning lines were put through the beak. We then adjusted another set of positioning lines in the plane with the second largest projection, which would also pass through two eyeballs. This was followed by the second positioning scan. After the second positioning scan, one standard sagittal plane and other symmetrical planes passing through the eyeballs were obtained. We then obtained standard sagittal brain slices by adjusting positioning lines to pass through the mid-line of the brain on the last positioning images. According to the atlas and standard sagittal slices, we finally produced standard coronal brain slices by placing the set of positioning lines (which were perpendicular to the line that was tangential to the dorsal surface of the parietal and frontal bones) onto the mid-sagittal brain slice.

### Measurement and analysis

**Fig. 4:**
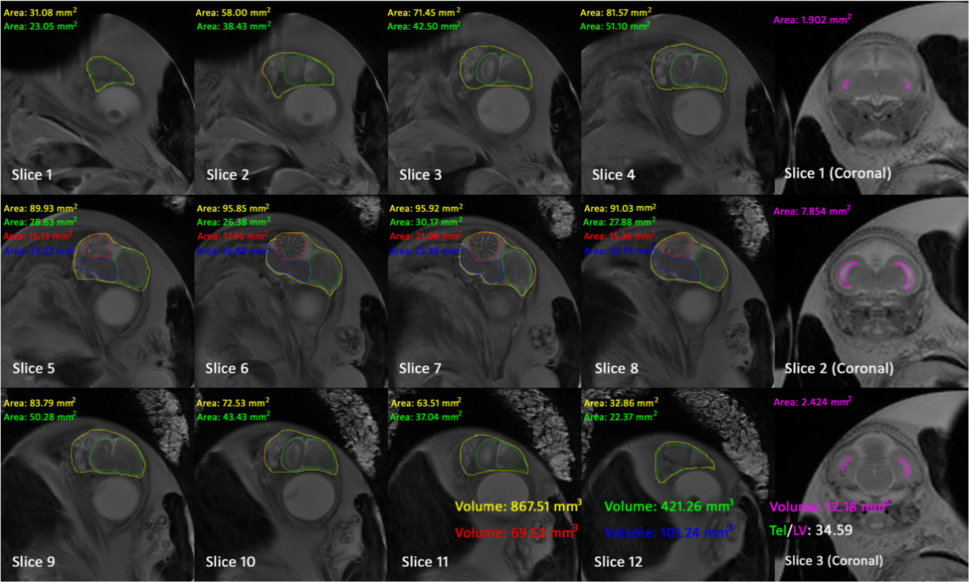
The brain segment result of a chick embryo at 17 days of incubation slice by slice in sagittal and coronal planes. The area of the whole brain (yellow), telencephalon (green), cerebellum (red), brainstem (blue) and LV (pink) were calculated by the ImageJ automatically. Since the slice thickness is 1 mm, the volume is the sum of each slice’s area, which is shown in the bottom of the figure

For volumetric analyses, the anatomical images (T2WI) were analyzed using the ImageJ software package. The telencephalon, cerebellum, brainstem, and whole brain volume were calculated from the sagittal brain slices, and LV volume was calculated from the coronal brain slices. The cortex was very thin, and the LV had not been completely formed during the early developmental stage (5 to 8 days of incubation); thus, we measured the telencephalic vesicle volume as a stand in for the LV volume. Different anatomical regions were manually outlined slice by slice according to a standard atlas. The software automatically calculated the segmented area of each brain region within each slice. Since slice thickness was 1 mm, the volume was equal to the sum of the segmented area within each slice. Figure 4 shows the brain segment results and corresponding areas within the sagittal and coronal slices of an embryo at 17 days of incubation. The whole brain, telencephalon, cerebellum, brainstem, and LV volume for each chick embryo were calculated from 5 to 20 days of incubation. To investigate the relationship between brain volume change and LV, the volume ratio from telencephalon to LV (Tel/LV) was calculated by dividing the telencephalon volume by the LV volume.

**Fig. 5:**
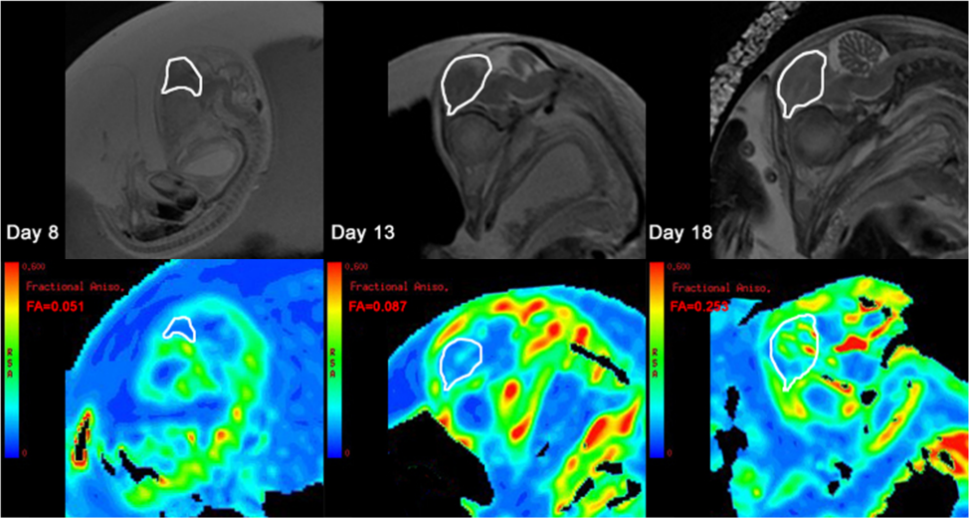
DTIs’ analysis of one embryo, imaged at 8, 13, 18 days of incubation. FA maps were automatically obtained using the built-in software in GE workstation (bottom line). Corresponding T2WIs (top line) were used to guide placement of ROIs in FA maps. The telencephalon was outlined as the ROI (white loop) and average FA was automatically calculated

DTIs were analyzed using a built-in software at a GE workstation, and FA maps were obtained. As shown in Fig. 5, the mid-sagittal brain slice was chosen, and an ROI was placed in the telencephalon region. T2WIs were used to assist with outlining the telencephalon. After drawing the ROI, the software calculated the average FA within the ROI. Volume data, Tel/LV ratio, and the FA value were all expressed as mean ± standard deviation (SD). The hatching rate was examined at 23 days of incubation.

## Abbreviations

BOLD: Blood oxygen level dependent
DTI: Diffusion tensor imaging
DWI: Diffusion weighted imaging
FA: Fractional anisotropy
FOV: Field of view
FSE: Fast spin echo
LV: Lateral ventricle
MRI: Magnetic resonance imaging
MRS: Magnetic resonance spectroscopy
ROI: Region of interest
SNR: Signal to noise ratio
Tel/LV: Volume ratio of telencephalon to lateral ventricle
2D: 2-dimensional
3D: 3-dimensional
